# The C:N:P:S stoichiometry of soil organic matter

**DOI:** 10.1007/s10533-016-0247-z

**Published:** 2016-09-23

**Authors:** Edward Tipping, Cayman J. Somerville, Jörg Luster

**Affiliations:** 1 Centre for Ecology and Hydrology, Lancaster Environment Centre, Lancaster, LA1 4AP UK; 2 Department of Marine and Environmental Science, Northeastern University, 365 Huntington Ave, Boston, MA 02115 USA; 3 Swiss Federal Research Institute for Forest, Snow and Landscape Research, 8903 Birmensdorf, Switzerland

**Keywords:** Carbon, Nitrogen, Phosphorus, Protein, Soil organic matter, Stoichiometry, Sulphur

## Abstract

The formation and turnover of soil organic matter (SOM) includes the biogeochemical processing of the macronutrient elements nitrogen (N), phosphorus (P) and sulphur (S), which alters their stoichiometric relationships to carbon (C) and to each other. We sought patterns among soil organic C, N, P and S in data for c. 2000 globally distributed soil samples, covering all soil horizons. For non-peat soils, strong negative correlations (p < 0.001) were found between N:C, P:C and S:C ratios and % organic carbon (OC), showing that SOM of soils with low OC concentrations (high in mineral matter) is rich in N, P and S. The results can be described approximately with a simple mixing model in which nutrient-poor SOM (NPSOM) has N:C, P:C and S:C ratios of 0.039, 0.0011 and 0.0054, while nutrient-rich SOM (NRSOM) has corresponding ratios of 0.12, 0.016 and 0.016, so that P is especially enriched in NRSOM compared to NPSOM. The trends hold across a range of ecosystems, for topsoils, including O horizons, and subsoils, and across different soil classes. The major exception is that tropical soils tend to have low P:C ratios especially at low N:C. We suggest that NRSOM comprises compounds selected by their strong adsorption to mineral matter. The stoichiometric patterns established here offer a new quantitative framework for SOM classification and characterisation, and provide important constraints to dynamic soil and ecosystem models of carbon turnover and nutrient dynamics.

**Electronic supplementary material:**

The online version of this article (doi:10.1007/s10533-016-0247-z) contains supplementary material, which is available to authorized users.

## Introduction

Soil organic matter (SOM) is a major global carbon pool and a key functional component of soils with respect to carbon and nutrient cycling, sorption processes, and soil physical properties including water retention. It is recognised to possess a range of turnover times, from less than one year to thousands of years (Amundson 2001), and to comprise complex chemical entities (Stevenson 1986; Kögel-Knabner 2002; Simpson and Simpson 2012). Whereas the chemical complexity was once thought to account for SOM stability, due to molecular recalcitrance, more recent thinking emphasises ecosystem properties notably sorptive protection and hindered microbial access (Schmidt et al. 2011; Dungait et al. 2012; Lehmann and Kleber 2015). The chemical structures of SOM have been elucidated principally through NMR spectroscopy (Baldock et al. 1992; Hatcher et al. 2001; Kögel-Knabner 2002; Simpson and Simpson 2012), while physical techniques have been used to study molecular size and aggregation (Wershaw 1999; Piccolo 2001). Radiocarbon provides information about turnover and age (Torn et al. 2009; Trumbore 2009; Mills et al. 2014). The N content of SOM (usually via the C:N ratio) is widely used to characterise SOM. However, the two other chief nutrients in SOM, phosphorus and sulphur, have received less attention, and it is possible that new insights about SOM could be gained by considering how their contents vary in different SOM types, and how the nutrient:C ratios vary with each other. Another reason to explore patterns in SOM nutrient elements is that SOM turnover is central to their ecosystem cycling (McGill and Cole 1981; Parton et al. 1987).

Over half a century ago, Walker and Adams (1958) showed that in New Zealand grassland soils, organic C, N, P and S were strongly related, both among different soils and with soil depth, and that the organic N:P ratio falls with depth, which led to the identification of the key role of P in ecosystem and soil development and function. Stevenson (1986) remarked that the composition of the “resistant humus fraction” of soil, by which he meant SOM but not plant and animal residues or microbial biomass, was remarkably similar for soils from different regions of the world, and suggested an average C:N:P:S stoichiometry equivalent to 108:8:1:1 (rounded values, by mass). Cleveland and Liptzin (2007) conducted a meta-analysis primarily aimed at understanding the elemental compositions of soil microbes, but including data on topsoils, for which they quoted a C:N:P stoichiometry of 72:6:1. However they used total rather than organic P concentrations in their data analysis, and so strictly speaking their derived stoichiometry is not that of SOM. The same applies to the studies of Tian et al. (2010), Li et al. (2012) and Xu et al. (2013). In their analysis of data obtained using the Hedley fractionation method, Yang and Post (2011) found that while C and N were strongly correlated across major soil orders, neither was strongly correlated with organic P, and they concluded that P was “decoupled” from C and N in highly weathered soils. Kirkby et al. (2011) concluded that SOM of mineral soil has an approximately constant stoichiometry, and from the data they collated for soils from 22 countries, we calculated a rounded stoichiometry of 52:5:1:1 (C:N:P:S).

Despite these research efforts, the stoichiometric analysis of soils data is incomplete, for four reasons. (1) The previous data collations and analyses did not cover the full range of available data for different soil types. In particular they focused on soils with relatively low C contents, which may have limited the possibility to draw general conclusions, and obscured broad trends. The New Zealand soils studied by Walker and colleagues were mostly under grassland. Kirkby et al. (2011) restricted their data analysis to either the higher-density fraction of soils, or to published data with low C:N ratios (no values greater than 16.5). Cleveland and Liptzin (2007) also focused solely on mineral soils, the majority of the samples having C values of 10 % or less. However, soils and soil horizons of most interest to terrestrial ecologists and biogeochemists are often topsoils, including O horizons, with comparatively low mineral contents (see e.g. Ågren et al. 2013; Hatton et al. 2015; Hobbie 2015), and it is therefore appropriate to include such soils in a wider stoichiometric analysis. (2) The trends with depth in NP ratio shown by Walker and Adams (1958) contradict the conclusion of Yang and Post (2011) that N and P are decoupled, neither do they fit with the notion that SOM has near-constant elemental composition. (3) These previous analyses considered the C, N, P and S contents of the soil as a whole rather than SOM, whereas a more informative approach might be to compare N:C, P:C and S:C ratios, which are direct measures of the element enrichment of organic matter. Manzoni et al. (2010) for example, in a meta-analysis of litter stoichiometry, constructed plots of C:P against C:N as a way to visualise the data, and thereby demonstrated a strong pattern, which extended to the data collated by Cleveland and Liptzin (2007) for soils. (4) The literature contains many more data on C:N:P:S stoichiometry than have so far been analysed together. For example the soil data sets of Cleveland and Liptzin (2007) and Yang and Post (2011) comprised only 142 and 178 samples respectively. That of Kirkby et al. (2011) was larger (>500 examples of C:P ratios), but was nonetheless only about 25 % of available data known to us.

Therefore, to improve understanding of the C:N:P:S stoichiometry of SOM, we conducted a new meta-analysis, making use of a greater amount of published data (for >2000 samples from 76 papers or reports), covering a wider range of soils, including soils from forest, grassland and arable land, together with peats, with data from all soil horizons (including O horizons), and exploring relationships between the stoichiometries of SOM and fresh litter (cf. Manzoni et al. 2010; Hobbie 2015). We focused on the element ratios of SOM rather than those of soil *per se*. We aimed to determine whether constant stoichiometry might apply to identifiable classes of SOM, whether there are systematic trends in SOM, and whether there are systematic differences in stoichiometric relationships between topsoils and subsoils, among soils differing in natural or semi-natural vegetation and land use, between temperate and tropical soils, and among major soil types. Such knowledge might shed light on how C, N, P and S are incorporated into SOM during the initial processing of plant litter, and subsequent microbial transformations, physical stabilisation and long-term turnover. In particular the results could foster the use of element stoichiometry to constrain conceptual and process-based models of SOM dynamics, and to link them to nutrient cycling models, permitting a more integrated approach to soil biogeochemistry.

## Collation and analysis of data

Data on the organic C, N, P and S concentrations of soil samples were collected mainly from the published literature, but also from some unpublished studies, a total of 76 sources (Table S1). The results were used as reported. Broadly the samples were of two kinds. About half referred to an identified soil horizon, principally O, A, E, B and C. The remainder had been taken between specified depths (e.g. 0–15, 45–60 cm) and in some cases would have been mixtures of material from more than one soil horizon, but in the absence of reported information about the horizons each was necessarily treated as a single sample for the analysis.

Where information about soil genesis was available, we divided the data by soil type. For this, we grouped the soils broadly according to the degree of soil development and prevailing soil forming processes, using the information given by the original source. This information was either a soil type according to a widely accepted classification system, or a horizonation showing diagnostic horizons. Thus, we differentiated weakly-developed soils (e.g. Rendzinas, Regosols, Inceptisols, Entisols), brown soils (mainly Cambisols, but also soils with SOM accumulation such as Chernozems), soils with clay translocation (e.g., Luvisols, Acrisols, Alisols), podzolized soils, hydromorphic soils, peat and highly weathered tropical soils without or with only weak argic horizons (Oxisols, Ferralsols). Because of the disturbance of natural soil formation by agricultural practice, soil types were assigned only to uncultivated soils.

For comparative purposes, we divided soils into topsoils and subsoils. Topsoils included samples to a depth of up to 30 cm when only a single depth was sampled. If several depths were taken the top one or two layers were counted as topsoils, to a depth of 10 cm. Where soils were sampled by horizon, O and A horizons were counted as topsoils.

In this work, we treat O horizons as soil. We acknowledge that some authors consider O horizon material to be litter, and some soil sampling conventions do not include O-horizon C in the total stock (e.g. Robertson and Paul 2000). We follow the FAO system in which O horizons are counted as soil, considered to be organic matter in various stages of decomposition (http://www.fao.org/docrep/w8594e/w8594e0g.htm; accessed May 2016). We apply the term litter to material recently shed by the plant (e.g. Trofymow et al. 1995), and not sampled as the O-horizon.

Only values for organic C and P were used, and only analyses of organic S, except for Swiss forest topsoils with high %C that could safely be assumed to contain only organic S. In a small minority of cases, organic N concentrations were reported, but most data referred to total N and this was the variable used in the meta-analysis, i.e. we assumed it was equal to organic N. According to Schulten and Schnitzer (1998) inorganic N is on average about 5 % of total soil N, but the proportion can be higher in deeper soil (Young and Aldag 1982). Stevenson (1986) compiled data for different soils to a depth of one metre and found inorganic nitrogen to average about 10 % of the total, although the proportion was very low in organic-rich horizons and peats. An average correction might be applied to improve the estimates of soil N concentration, but the most commonly reported stoichiometric ratio, C:N, is usually based on total N and we followed this “convention”. Thus, throughout the following text, element abbreviations refer to what are assumed to be organic forms.

We restricted our data analysis to soils for which at least two of N, P and S were reported, because of the need to compare contents of the elements in SOM. In the great majority of cases, the reports included soil concentrations of the elements, but in a minority only element ratios were available. In the cases of peat samples with no reported C concentrations, but which could be assumed to comprise almost entirely organic matter, we assumed the C concentration to be 50 %.

The collated data were obtained with a variety of analytical techniques, and we accepted the authors’ judgements about their efficacy. However, the determination of organic P is subject to uncertainty and there are a number of methods (Turner et al. 2005; Olsen and Sommers 1982). We identified two broad approaches. The first was estimation of organic P as the difference between total and inorganic P, the former often being obtained by extraction with acid after ashing and the latter without ashing. The second covers methods that involve extraction with base, notably the Hedley fractionation scheme (Hedley and Stewart 1982).

We chose to present results primarily as the mass ratios N:C, P:C and S:C rather than the more commonly used C:N, C:P and C:S, because we wanted to focus on the enrichment of N, P and S in SOM. Thus N:C, P:C and S:C are essentially concentrations of N, P and S in the organic matter, since the C content of SOM is roughly constant at about 50 %. But at key points we also report C:N, C:P and C:S ratios in an effort to maximise clarity. Following the work of Cleveland and Liptzin (2007) on soils and microbes, and Manzoni et al. (2010) on litter and soils, we used logarithmic scales to even the spread of data, cope with data scatter, and avoid bias associated with high element concentrations. We performed statistical tests using R (R Core Team 2013), and modelling with Microsoft Excel.

## Results

We first consider all soils except ombrotrophic peats, which are treated separately afterwards because they obtain P from external, principally atmospheric, sources (Tipping et al. 2014) rather than from local weathering. The results for non-peat soils covered topsoils and subsoils, different vegetation and soil types and land uses, and were divided according to temperate and tropical climates (Table 1). There were 1710 non-peat soil samples in total, 892 of which had been sampled according to pedogenetic soil horizon, and these were divided as follows: O, 89 samples (10 %); A 439 (49 %); B 214 (24 %); C, 95 (11 %); E, 26 (3 %); G 29 (3 %). Over all 1710 samples the C concentrations ranged from 0.06 to 60.5 %, with 10th, 50th and 90th percentile values of 0.5, 2.4 and 12.3 % respectively.

**Table 1:** Summary of data for samples that could be classified by vegetation type

Ecosystem	Climate	Samples with N & P		Samples with N & S	
		Topsoils	Subsoils	Topsoils	Subsoils
Arable	Temperate	152	39	65	16
	Tropical	17		12	15
Forest	Temperate	311	170	82	28
	Tropical	114	14	3	7
Grassland	Temperate	329	214	97	20
	Tropical	51	14		
Peatland	Temperate	257	5	17	
	Tropical	34			
Shrub	Temperate	24	27		

The geographical distribution of samples was: Africa 6 %, Asia 3 %, Europe 41 %, N America 21 %, Oceania 22 %, S & C America 7 %

We compared results from the two broad analytical methods for organic P, i.e. difference and extraction (see “Collation and analysis of data” section), in terms of P:C relationships. For both methods, %P was found to increase significantly with %C, but the %P values obtained by difference tended to fall above the central trend, while the extraction values tended to fall below it (Fig. S1). This was as expected, since the difference method may underestimate inorganic P and thereby overestimate organic P, while extraction may fail to release all the organic P and thereby underestimate it. Probably neither approach can be considered to be more correct (Turner et al. 2005), and therefore we conducted our meta-analysis combining data from both methods.

**Fig. 1:**
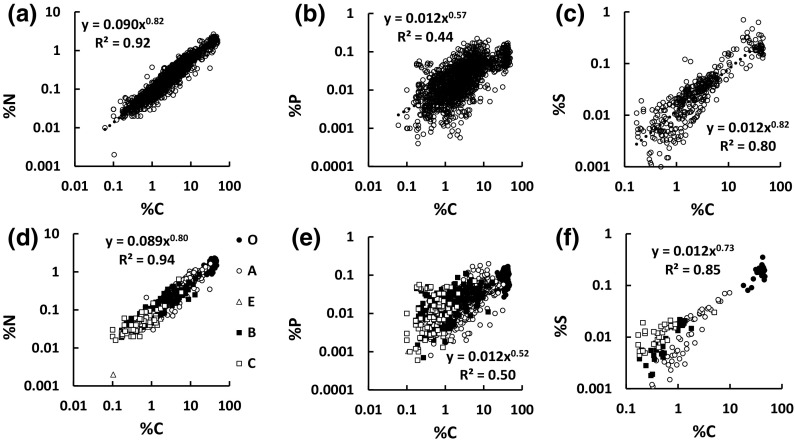
Regressions of %N versus %C, %P versus %C, % S versus %C for all soils other than ombrotrophic peats. All trends are significant (p < 0.001). Panels **a**–**c** show all data, panels **d**–**f** show data for samples with identified soil horizons. The numbers of data per horizon (O, A, E, B, C) are: 86, 439, 26, 214, 95 for %N versus %C (panel **d**); 85, 414, 26, 212, 94 for %P versus %C (panel **e**); 38, 55, 0, 30, 28 for %S versus %C (panel **f**)

Power-law regressions of %N (1666 points), %P (1452 points) and %S (378 points) against %C for all non-peat soils with available data show highly significant linear relationships (Fig. 1a–c). A common feature of the plots is that the exponent is significantly (p < 0.001) less than one in each case, c. 0.8 for N and S, and c. 0.6 for P, which means that the N:C, P:C and S:C ratios vary with %C, increasing significantly and continuously with decreasing %C, and with increasing mineral matter. Therefore there cannot be a single C:N:P:S stoichiometry common to all SOM. The lower exponent for P in Fig. 1 means that the P:C ratio is more variable among the soils than the N:C and S:C ratios. This is also seen through cumulative distribution plots (Fig. S2) which show lognormal behaviour with standard deviations increasing in the order N < S < P. Thus the relative variation of the P content of SOM is greater than that of S, which is in turn greater than that of N.

The Fig. 1 panels (d)–(f) show results only for samples taken from a single identified soil horizon, with 860, 831 and 151 data for %N, %P and %S respectively versus %C. The power-law exponents remain significantly (p < 0.001) less than one in each case. Compared to the results for the complete data sets, there are three significant differences among the six power-law constants and exponents; the constant for %N versus %C is lower (p < 0.001), the exponent for %P versus %C is lower (p < 0.05), as is that for %S versus %C (p < 0.05). However, even though they are significant, the differences are small and do not detract from the strong broad trends that emerge from the plots in Fig. 1. The plots reveal how data for the different horizons fall into broad groups, from O (top right), through A, E and B, to C (bottom left), although with considerable overlap. If data for O horizons are excluded (86, 85, 38 data respectively for the plots of %N, %P and %S vs %C), as might be done if these were regarded not as soil but as litter (see see “Collation and analysis of data” section), none of the constants or intercepts differ significantly (p > 0.2) from those obtained using all the data for samples from identified horizons. This demonstrates that the trends established from these plots do not result from combining data for high %C O horizons with those for low %C mineral soils; the trends persist across the entire range of soil %C values.

**Fig. 2:**
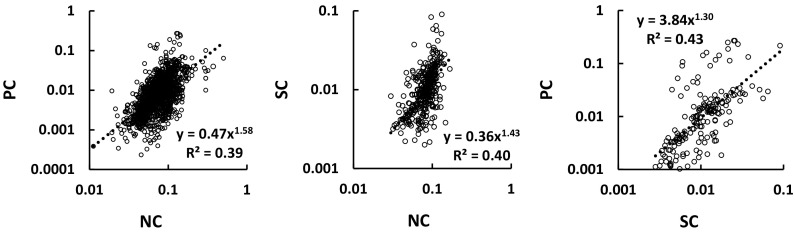
Element ratios plotted against each other for all soils other than ombrotrophic peats. All trends are significant (p < 0.001)

By plotting ratios of N:C, P:C and S:C against each other we can see how strong their interrelationships are, in other words whether enrichments in one element parallel those in another. Because the C content of SOM does not vary greatly (it is around 50 %) the ratios are essentially measures of the concentrations of the other 3 elements in SOM. The logarithmic relationships are indeed highly significant (Fig. 2). There are outliers at high N:C and high P:C, nearly all associated with samples having low %C (<0.5) which might have rendered the analyses less reliable.

The plotting of ratios involving a common variable can lead to spurious correlations, i.e. apparent relationships can be obtained even though there is no true underlying interdependence of the variables (Aitchison 1986). We looked into this by randomly choosing values from within the ranges of observations of C, N, P and S and constructing plots with the same variables as those in Fig. 2. Significant correlations were indeed obtained (Fig. S3), but the relationships were quite different from those of Fig. 2. In particular, the slopes were substantially less than unity (c. 0.5), whereas the observed ones are greater, and the R^2^ values were lower (c. 0.25). Therefore the relationships of Fig. 2 can be accepted as real.

**Fig. 3:**
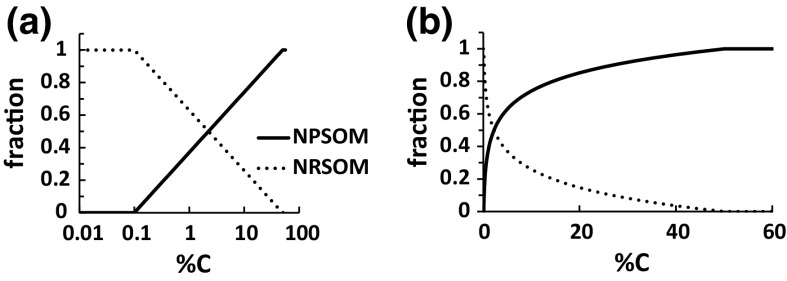
Schematic of the mixing model, logarithmic (panel **a**) and linear (panel **b**) versions. The *y*-axis is the fraction of NPSOM or NRSOM

The results in Figs. 1 and 2 suggest that SOM stoichiometry can be represented with a simple mixing model in which any sample of SOM comprises two end-members, one nutrient-poor (NPSOM) and one nutrient-rich (NRSOM). To parameterise the model we assumed that the end-member %C values were 0.1 % or less (at or below this value, all SOM is NRSOM) and 50 % or greater (at or above this value all SOM is NPSOM). The fraction of NPSOM (F_NPSOM_) was assumed to increase linearly with log %C (Fig. 3). Formally:

$$ \%{\text{C}} \;< \;0.1\quad{\text{F}}_{\text{NPSOM}} = 0\quad{\text{F}}_{\text{NRSOM}} = 1 $$

$$ 0.1 < \;\% {\text{C}} <\; 50$$

$${\text{F}}_{\text{NPSOM}} = \log_{10} {{\left( {\% {\text{C}}/0.1} \right)} \mathord{\left/ {\vphantom {{\left( {\% {\text{C}}/0.1} \right)} {\log_{10} \left( {50/0.1} \right)}}} \right. \kern-0pt} {\log_{10} \left( {50/0.1} \right)}}$$

$${\text{F}}_{\text{NRSOM}} = 1{-}{\text{F}}_{\text{NPSOM}}$$

$$ \% {\text{C}} > 50\quad{\text{F}}_{\text{NPSOM}} = 1\quad{\text{F}}_{\text{NRSOM}} = 0 $$

**Fig. 4:**
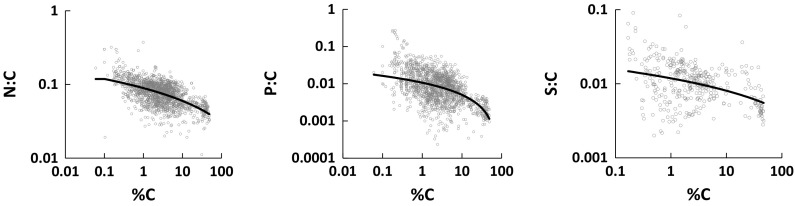
Element ratios to C versus %C for all soils other than ombrotrophic peats, fitted with the two-endmember mixing model. The left end of each solid line corresponds to NRSOM, the right end to NPSOM (see Fig. 3)

The end-member N:C, P:C and S:C values were fitted by minimizing the sum of squared residuals in log %N, log %P and log %S. Figure 4 compares observed and modelled variations of the N:C, P:C and S:C ratios with %C. The end-member compositions are summarised in Table 2, and show that NRSOM has three times the N, 15 times the P, and three times the S as NPSOM. The (rounded) stoichiometries of the end-members can be expressed in terms of C:N:P:S as 919:36:1:5 for NPSOM and 61:7:1:1 for NRSOM.

**Table 2:** Mixing model parameters

	NPSOM	NRSOM
%C for 100%	≥50	≤0.1
N:C	0.039	0.12
P:C	0.0011	0.016
S:C	0.0054	0.016
C:N	25	8.4
C:P	919	61
C:S	185	64

Ratios in g g^−1^

We performed simulations with the parameterized model to test whether the mixing of soil horizons, which may occur when sampling is done by depth rather than by horizon, influences the patterns shown in Figs. 1, 2 and 4. Mixing caused some systematic deviations; %N and %P values in mixed samples fell below the central trend of Fig. 1, while N:C and P:C values fell below the model trend in Fig. 4. However there was no deviation from the modelled P:C versus N:C trend of Fig. 2. Inspection of data for samples from identified horizons showed that the largest deviations would occur for forest soils with O horizons overlying mineral horizons (mostly A, some E). Taking results for Swiss forest soils (data set SD_07 in Table S1), the most extreme differences in C concentration are found for an O horizon with c. 35 %C and an underlying mineral horizon with c. 1 %C. More typically, the concentrations are 35 and 5 %C respectively. We simulated these two cases using the model (Fig. S4). The results for the first pair of horizons show appreciable deviations for the modelled line, although within the data scatter, whereas those for the second pair show only small deviations. Since forest soils only account for 26 % of the data when sampling was done by depth intervals, and since in forests the 35:1 %C case is extreme, we conclude that there will have been few cases with large deviations from the model, and so the mixing effect will have had little overall influence on our analysis.

**Fig. 5:**
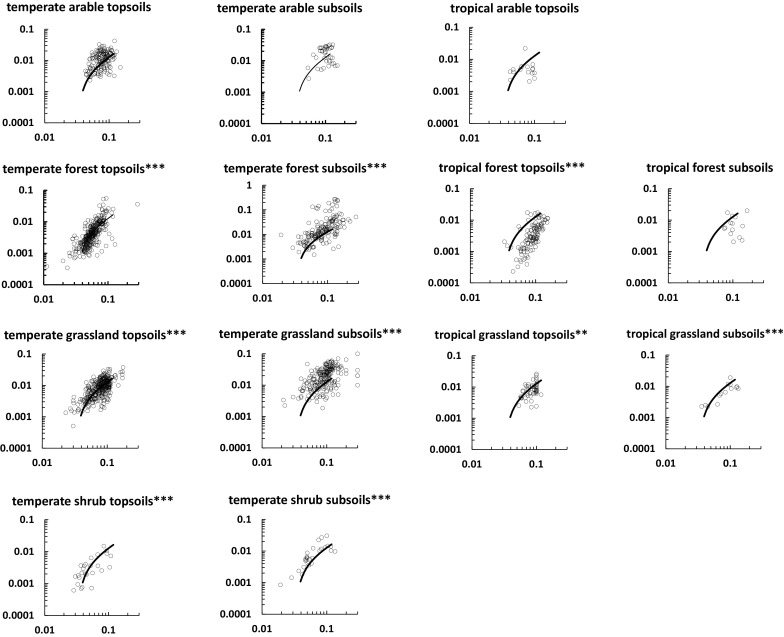
Relationships between P:C (*y*-axis) and N:C (*x*-axis) for soils from different ecosystems. The significance indicators refer to power-law regressions; *p < 0.05, **p < 0.01, ***p < 0.001. The *solid line* shows the mixing model trend. Axis labels are omitted for clarity

**Fig. 6:**
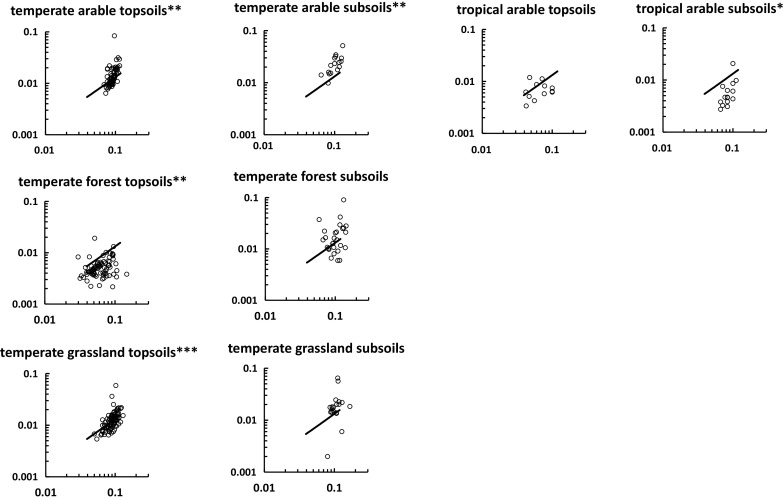
Relationships between S:C (*y*-axis) and N:C (*x*-axis) for soils from different ecosystems. The significance indicators refer to power-law regressions; *p < 0.05, **p < 0.01, ***p < 0.001. The *solid line* shows the mixing model trend. Axis labels are omitted for clarity

We used plots of P:C or S:C versus N:C to show results for different ecosystems categorized in terms of vegetation or land use and climate (Figs. 5, 6). Power law regression (which gives straight lines when plotted logarithmically) was used to judge the significance of P:C or S:C versus N:C relationships. The mixing model, which gives a curved logarithmic plot, was used as a yardstick against which results for different ecosystems can be judged (Figs. 5, 6). Fourteen of the 21 plots in Figs. 5 and 6 (i.e. 67 %) show significant power law regressions. No significant regressions are found for P:C versus N:C in arable soils (Fig. 5), perhaps because the data ranges are relatively small, as a consequence of fertilizer application. Otherwise non-significant regressions are found for ecosystems or land-uses that yield few points. The major systematic deviation from the model is shown by tropical forest soils for P:C versus N:C (Fig. 5), for which the main difference arises from the NPSOM stoichiometry, since the points trend towards the default NRSOM end-member stoichiometry. This might reflect the lower P content of tropical forest litter (see below). The S:C versus N:C plots for tropical arable and temperate forest also tend to fall below the model trend (Fig. 6). A feature of the plots is that subsoil N:C, P:C and S:C ratios tend to be higher for subsoils (except temperate arable) which reflects their generally lower C concentrations.

**Fig. 7:**
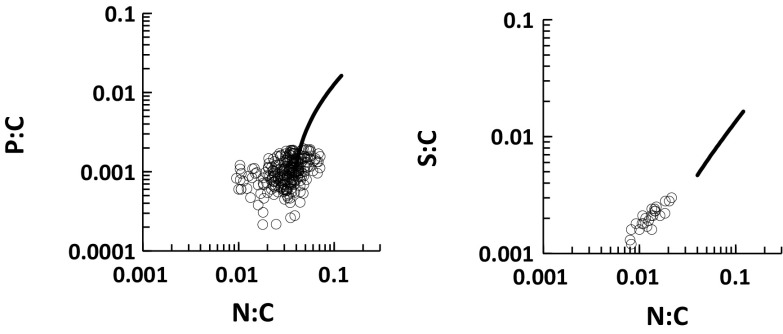
Relationships between P:C and N:C, and between S:C and N:C, for ombrotrophic peat topsoils

Data for ombrotrophic peats do not follow the mixing model, with nearly all samples showing lower N:C, P:C and S:C ratios than the NPSOM end-member (Fig. 7). In other words there is little overlap of peat stoichiometries with those of other soils. Therefore peats are best treated as separate entities.

Plots of P:C or S:C versus N:C were also made for different major soil classes (Figs. S5, S6). No major deviations from the model were found in the P:C versus N:C relationships for temperate soils, or in any of the S:C versus N:C relationships. However, tropical soils showed similar patterns to those for tropical forests in Fig. 5, i.e. the P:C ratios tended to fall below the model line, especially at low N:C. We cannot be sure that this is a general feature of tropical soils, or whether it arises because the data set is relatively small, with many of the data referring to South American forest soils. We attempted to fit the mixing model separately for tropical soils, but the data were too few to obtain reliable values for the NPSOM P:C and S:C ratios.

**Fig. 8:**
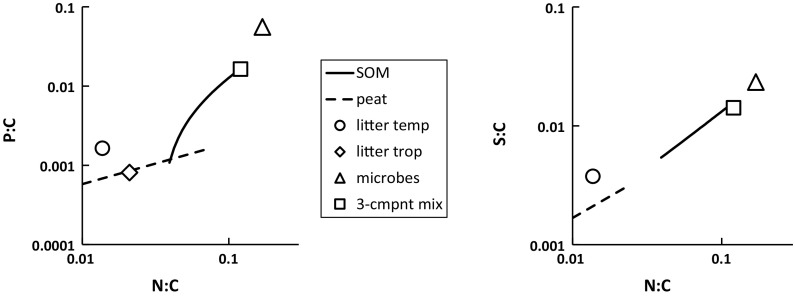
Overall picture of C, N, S and P in SOM. Data for temperate litter stoichiometry are from Trofymow et al. (1995), for tropical plant (tree) litter from Tripathi and Singh (1992), Thompson and Vitousek (1997), Chuyong et al. (2002), Hirobe et al. (2004) and Isaac and Nair (2005), and for microbial biomass from Fagerbakke et al. (1996), Cleveland and Liptzin (2007), Griffiths et al. (2012). The illustrative three-component mixture has a stoichiometry adjusted to coincide with that of NRSOM (see text)

We combined the mixing model output with stoichiometry data from the literature for plant litter and microbial biomass to obtain an overall picture of C:N:P:S stoichiometry in litter-soil systems (Fig. 8), following the plotting approach of Manzoni et al. (2010). We see that the stoichiometry of the NRSOM end member (top right end of the lines) is quite close to that of microbes. The plots show that NPSOM (bottom left end of the lines) has P:C and S:C ratios similar to those of fresh litter, but the N:C ratio is higher, the litter value being only 0.016, about 40 % of the NPSOM value (litter C:N = 63). Peat N:C values are similar to those of plant litter. For illustration, we also show results for a hypothetical three-component mixture comprising 35 % average protein (composition from Satyanarayana and Chakrapani 2006) 2.8 % phytic acid, and 62 % a compound with C but no N, P or S. This mixture composition was chosen to produce N:C, P:C and S:C ratios close to those of the NRSOM end-member.

**Fig. 9:**
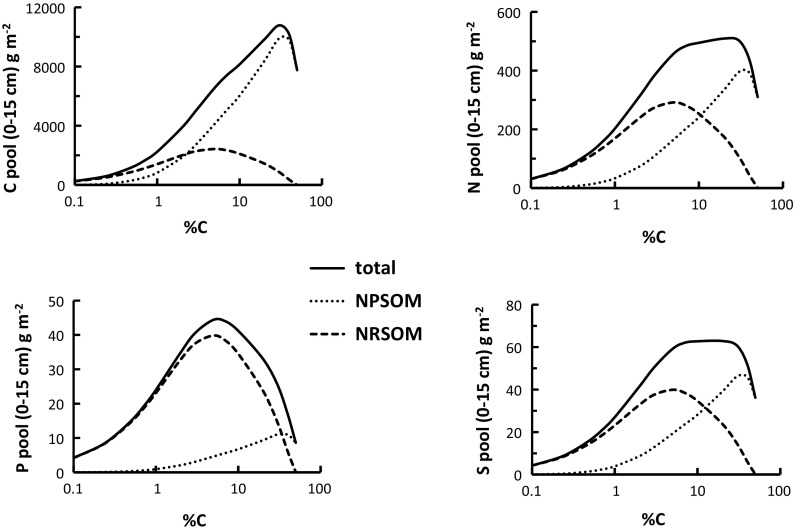
Variation of pool sizes of organic C, N, P and S with soil carbon concentration, calculated from the NPSOM–NRSOM mixing model and an assumed dependence of bulk density on %C (see text). The decreases in the NPSOM and total pools of C (*top left panel*) arise because of the modelled dependence of bulk density on %C

The mixing model can be combined with values of soil bulk density, estimated from %C, to construct plots that show how soil C, N, P and S pools vary with soil %C. We used a relationship of bulk density (BD) to soil %C established for UK soils (BD = 1.29e^−0.206 %C^ + 2.51e^−0.003 %C^ − 2.057; Reynolds et al. 2013), but very similar results were obtained with an alternative formula (BD = 1.83e^−0.121 OC^0.5^ where OC is in g kg^−1^; Alexander 1989). The calculations were performed for a soil depth interval of 15 cm (Fig. 9), but the trends are independent of the choice of depth interval. Stones were assumed absent. The results show that the NPSOM and NRSOM contents are equal, in terms of C, when the soil C content is 2.2 %. However, this does not apply to the distributions of N, P and S. For N the amounts are equal when %C is about 10 %, for P the equivalence point is 40 % and for S it is 20 %. Another feature is that the C pool increases with %C to quite high %C, whereas it is fairly steady for both N and S in the range 3–20 %C, in all three cases falling away at the highest %C (although this is very sensitive to the bulk density values). The P pool shows a well-defined and quite sharp maximum at 5 %C. The plots emphasise the role of NRSOM in accounting for N, P and S contents, showing the similarity of N and S and the strong association of P with the NRSOM fraction.

## Discussion

Our results show that N:C, P:C and S:C ratios vary systematically across all temperate soils, following a stoichiometric continuum. Each ratio increases with decreasing C concentration, irrespective of soil horizon, soil type and vegetation type or land use. Tropical soils follow similar trends but with lower P:C ratios. Considering the whole range of soil C concentrations, there is clearly not a constant or typical stoichiometric composition of SOM, but it can be seen how a constant composition might appear to apply if relatively few data for only limited ranges of soils are considered, as in the work of Stevenson (1986), Cleveland and Liptzin (2007) and Kirkby et al. (2011). The constant compositions proposed by these authors (see “Introduction” section) are quite similar to that of NRSOM, but less rich in N, P and S.

According to the mixing model, the stoichiometry of NPSOM corresponds to that of average litter for P:C and S:C, while its N:C ratio is somewhat higher (Fig. 8). Therefore, on average in soils other than peats, the initial stages of decomposition, and the formation of NPSOM, involve approximately proportional losses of C, P and S, but there is a smaller relative loss of N. Temperate soils tend to have higher P:C ratios than tropical ones (Figs. 5, S2), probably reflecting the greater extent of weathering and consequent lower P availability of the latter. From the results in Fig. 7 we find that ombrotrophic peat SOM stoichiometry differs from that of NPSOM, being appreciably more nutrient depleted, which may arise because such peats are intrinsically low in nutrients and so effectively give rise to a second type of NPSOM, having lower N:C, P:C and S:C ratios than those derived for non-peat soils from the mixing model (Fig. 8). Therefore to cover all the soil samples analysed in this work, we distinguish ombrotrophic peat SOM from that of the non-peat soils, and the remaining discussion focuses on the non-peat SOM.

The NRSOM stoichiometry (Table 2) is quite similar to that of microbial biomass (Fig. 8), and some of the NRSOM must actually be microbial biomass that was alive at the time of sampling, although it is unlikely to be a major part because living microbial biomass is only a few per cent of total SOM (Jenkinson and Ladd 1981; Kassim et al. 1981; Stevenson 1994). Kirkby et al. (2011) proposed that the similar stoichiometry of the stable portion of SOM to microbes indicates that it is comprised of microbial detritus, and suggested that microbial biomass is the immediate precursor of the stable SOM, while plant biomass is the penultimate precursor. However, it is difficult to envisage a mechanism whereby little-changed microbial biomass could be preserved so as to create the NRSOM pool. It seems more likely that some individual molecules released on the decomposition of microbial biomass or plant litter are selectively incorporated into the NRSOM fraction.

Protein is a likely major source of the nitrogen in NRSOM. It is well-known that a high proportion of the N in material isolated from soils by alkaline extraction is derived from proteins (Stevenson 1986; Schulten and Schnitzer 1998; Knicker 2004, 2011), and a significant role for proteinaceous material in the formation of stable SOM has been advanced by a number of authors (Amelung 2003; Rumpel et al. 2004; Kleber et al. 2007; Rillig et al. 2007; Knicker 2011). Our illustrative three-component mixture of NRSOM (Fig. 8) comprises 35 % protein, which agrees with the estimate (34 %) by Knicker (2011) for SOM with an N:C ratio of 0.1, and also the range of values (26–36 %) estimated by Cusack et al. (2013) from NMR data for cultivated Hawaiian soils with N:C ~0.09. The proximate source of protein-derived N in NRSOM cannot be deduced from its stoichiometry, so it could be plant or microbial protein or both. Rillig et al. (2007) stated that microbial proteins are thought to be the more persistent in soil, but that this was not yet proven.

Proteinaceous material might also furnish NRSOM with a significant amount of S, in the amino acids cysteine and methionine, and indeed the assumption of average protein stoichiometry in the three-component mixture yields a reasonable estimate of the S content of NRSOM (Fig. 8). However, data for a range of soils (Autry and Fitzgerald 1990; Zhao et al. 1996) show that only about one-fifth of the S in SOM can be ascribed to amino acids (as organic S reducible with Raney nickel). Moreover, if protein were the main form of organic S in NRSOM, it would be expected that the fraction of amino acid S would increase with the S:C ratio. However, analysis of data in references from our database, combined with the results of Autry and Fitzgerald (1990), showed this not to be the case; the proportions of the three principal forms of organic S, i.e. ester-sulphates, amino acids and C-S bonded S not in amino acids (chiefly sulphonates) showed no significant variation with S:C ratio.

Although proteins do not contain P in their primary structures, post-translational phosphorylation may occur (see e.g. Cohen 2000), including in bacteria (Deutscher and Saier 2005), and this could result in the co-occurrence of P with N and S in NRSOM. However, according to Dalal (1977) and Turner et al. (2002), the dominant class of organic P in soils comprises inositol phosphates, principally derived from plants but also formed by microbes. *Myo*-inositol hexakisphosphate (IP_6_, phytic acid) is the most prevalent form and this might therefore account for much of the P in NRSOM, if it is assumed that it comprises about 3 % of the total mass as in the illustrative three-component mixture (see “Results” section). However, to attribute all of the P in NRSOM to IP_6_ is likely too much of a simplification, since reasons for variation in the IP_6_ contents of soils remain elusive (Turner et al. 2012; Jørgensen et al. 2015).

The dominance of NRSOM over NPSOM when the mineral:SOM ratio is high, i.e. %C is low (Fig. 3), occurs under conditions in which adsorption is most likely strongest, suggesting adsorption as the mechanism by which NRSOM accumulates. This is consistent with the high-density (mineral-rich) fraction of SOM usually having low C:N ratios (Baisden et al. 2002; Sollins et al. 2006; von Lützow et al. 2007; Schrumpf et al. 2013), and the known strong adsorption by mineral surfaces of proteins (Kleber et al. 2007) and phytic acid (Anderson et al. 1974; De Groot and Golterman 1993; Celi and Barberis 2006). Adsorption by mineral matter is thought to stabilise SOM by rendering it less accessible to microbial attack (von Lützow et al. 2006; Schmidt et al. 2011; Kleber et al. 2011). Therefore if adsorbed NRSOM is strongly stabilised, while the decomposition rate of NPSOM is relatively high, greater accumulation of NRSOM can occur even if the input rate of NPSOM to the soil horizon exceeds that of NRSOM. This would lead to a high NRSOM:NPSOM ratio, as shown in Fig. 3 for C concentrations <1 %. But under circumstances where the sorption of NRSOM is weak, and stabilisation therefore less, then the preferential accumulation of NRSOM will be reduced, or not occur at all. This would be expected when the mineral:SOM ratio is low (high %C), as in Fig. 3 for C concentrations >20 %. In between these adsorption extremes, there is a transition from NRSOM to NPSOM dominance, with a crossover point at a C concentration of about 2 % (Fig. 3). If this adsorption mechanism is correct, then NRSOM is a highly selected fraction of SOM that has built up over a relatively long period of time, compared to the NPSOM. Therefore there should be a positive correlation between radiocarbon age and the N:C, P:C and S:C ratios, and this has indeed been demonstrated for N:C by Rumpel and Kögel-Knabner (2011) using data for farmed soils in England. The generally observed increases with soil depth of both N:C ratio (Batjes 1996) and radiocarbon age (Scharpenseel 1993) also fit this expected behaviour.

The NRSOM may contain strongly-sorbing molecules that are rich in one of the elements (e.g. phytic acid in the case of P), or two them (e.g. un-phosphorylated proteins for N and S) or all three (e.g. phosphorylated proteins). Our results do not rule in or out the possibility that some NRSOM components are “humic substances”, i.e. (bio)synthetic products of plant and microbial decomposition, which may also contain one, two or three of the elements. Recent publications by Schmidt et al. (2011) and Lehmann and Kleber (2015) question whether “humification” actually occurs, and according to Kelleher and Simpson (2006) all organic components of the “humic substances” extracted by base are recognisable biochemicals. But the important point from our meta-analysis is that the NRSOM likely comprises molecules selected by the soil system for their ability to sorb strongly to mineral matter. The high N:C, P:C and S:C ratios of this material mean that molecules containing one or more of the three nutrient elements, especially P, tend to adsorb more strongly than SOM as a whole, but it is quite possible, indeed likely, that some of the NRSOM fraction comprises molecules not containing N, P or S, as illustrated by the three-component mixture of Fig. 8.

The arguments presented above address the stabilisation and accumulation of NRSOM via preferential adsorption, which probably applies widely to well-drained soils. However, carbon low in ^14^C, and therefore aged, is also found in poorly-drained soils with high C concentrations that are temporarily or permanently anoxic, and where NPSOM may dominate. The low-nutrient organic matter buried in peats is the obvious example of the preservation of old C due to the lack of oxygen for decomposition, but such SOM longevity may also arise in gleys and other soils in which pockets of anoxia can develop (Hall et al. 2015). This might also explain the presence of organic C with low N:C ratios and depleted in radiocarbon (Δ^14^C in the range 0 to −100 ‰) in occluded low density fractions, reported by Schrumpf et al. (2013). Thus, SOM can be stabilised by both adsorptive stabilisation and anoxic preservation in aggregates, and presumably there can be intermediate conditions in which stability arises from both mechanisms.

Although our results demonstrate strong and highly significant trends there is also much data scatter, which could arise for a number of reasons. Relative input rates of N, P and S may vary. The types of adsorbing organic molecules that comprise NRSOM may vary among plant types and microbial populations. There is considerable scope for variation in the adsorption process itself, because of differences in solution chemistry (including pH), the nature of the mineral surfaces, and particle size. There may be different combinations of the sorption and anoxia storage mechanisms for SOM. Soils differ in water permeability and thus in the downward transport and subsequent retention and modification of dissolved organic matter. There will inevitably be scatter due to analytical error, which may be greater for S and P simply because they are present in SOM at lower concentrations than N. Concentrations of organic N may be overestimated to different extents, because some N is present in inorganic forms. The high degree of scatter for P might reflect the use of different analytical methods, or by its presence in only a small proportion of the organic molecules, making it less strongly correlated to bulk C compared to N and perhaps also S. Scatter does not appear to arise simply from variations among ecosystem or soil types (Figs. 5, 6, S2, S3), except for differences between temperate and tropical systems. It may be fruitful to consider the remaining variation in terms of residual differences to model predictions, i.e. by using the soil C concentration to predict N, P and S concentrations in a given soil, and then seeking explanations for deviations.

The stoichiometric trends identified in this work provide significant constraints to ecosystem and soil models of carbon dynamics and nutrient cycling. The likely connection between C:N:P:S stoichiometry and the adsorption behaviour of SOM can be taken account of in dynamic modelling, and may fit well with the “Soil Continuum Model” conceptual approach advocated by Lehmann and Kleber (2015), which focuses on the processes, including adsorption and aggregation, that generate SOM. Improved models of SOM dynamics need to account quantitatively for pool sizes, SOM concentrations, radiocarbon ages, and C:N:P:S stoichiometry, and how they vary among soil types and horizons.

## Conclusions

- The three nutrient elements (N, P, S) display parallel enrichments in SOM, providing evidence for systematic stoichiometric behaviour, although with substantial scatter even when the data are plotted logarithmically.
- For non-peat soils, strong negative correlations (p < 0.001) were found between N:C, P:C and S:C ratios and % organic carbon (OC), showing that SOM of soils with low OC concentrations (high mineral matter) is enriched in N, P and S, with especially marked enrichment of P.
- The results conform to a simple end-member mixing model with one form of SOM that is nutrient-poor (NPSOM) and another that is nutrient-rich (NRSOM). Their relative amounts are predictable from the soil organic C concentration, such that NPSOM dominates when %C is high and NRSOM dominates when %C is low.
- The data show no major differences in P:C versus N:C and S:C versus N:C relationships amongst temperate ecosystems and soils. The NPSOM of tropical soils appears to have a lower P:C ratio than that of temperate soils.
- Ombrotrophic peats fall into a separate category from the NPSOM end-member, having lower N:C, P:C and S:C ratios.
- The NRSOM is created by the preferential adsorption by soil mineral matter of compounds rich in N, P and S.
- The stoichiometric patterns established in this work provide a new quantitative framework for SOM classification and characterisation.

## Electronic supplementary material

Below is the link to the electronic supplementary material.

Supplementary material 1 (DOCX 750 kb)

Supplementary material 2 (XLSX 276 kb)
