# Supplementary material for: The C:N:P:S stoichiometry of soil organic matter
Source: Biogeochemistry. 2016 Sep 23;130(1):117–31. doi: 10.1007/s10533-016-0247-z (PMC7175710; doi:10.1007/s10533-016-0247-z)
Supplement: Supplementary file 1 — Supplementary material 1 (DOCX 750 kb) [file 10533_2016_247_MOESM1_ESM.docx]

**The C-N-P-S stoichiometry of soil organic matter**

Edward Tipping, Cayman J Somerville, Jörg Luster

Supplementary figures

Figure S1. Relationships between soil organic phosphorus and soil organic carbon. The left-hand plot (n =946) shows results obtained by the difference between total and inorganic P, the right-hand plot (n = 504) is for results obtained by extractions with base. In each case the same power law relationship, fitted to the entire data set, is shown.

Figure S2. Log-normally distributed organic NC, PC and SC ratios for non-peat soils.

Figure S3. Spurious correlations between PC and NC, SC and NC and PC and SC, generated from randomly-chosen values within the ranges of %C, %N, %P and %S in the full data set. In each case 2000 points are plotted, and power-law fits and R^2^ values are shown. Note that these relationships are quite different from those in Figure 2.

We repeated this 10 times with different sets of random choices, and obtained:

PC vs NC slopes in the range: 0.44 - 0.54, R^2^ 0.21 - 0.29

SC vs NC slopes in the range: 0.48 - 0.54, R^2^ 0.23 - 0.28

PC vs SC slopes in the range: 0.47 - 0.57, R^2^ 0.24 - 0.31

1 %C & 35 %C 5 %C & 35 %C

%P

%N

%P

%N

P:C

N:C

P:C

N:C

Figure S4. Simulating the effects of horizon mixing when sampling according to pre-determined depth. The dotted lines show results from the fitted mixing model. The full lines show simulated results obtained by assuming that soil samples with different %C, and therefore different NPSOM and NRSOM contents, were mixed in different proportions. The NPSOM and NRSOM end-members had the stoichiometries shown in Table 2.

**Temperate weakly-developed soils**

Figure S5. PC vs NC for different soil types. The line in each case is from the NPSOM-NRSOM mixing model.


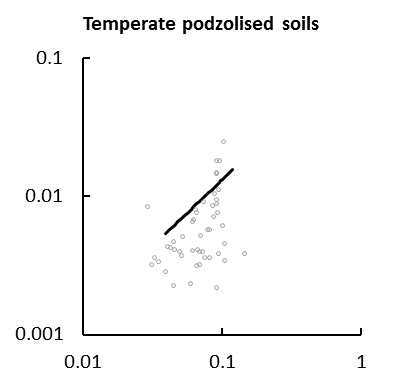


**Temperate weakly-developed soils**

**Tropical weakly-developed soils**

Figure S6. SC s NC for different soil types. The line in each case is from the NPSOM-NRSOM mixing model.
